# A Masquerade of Infectious Myositis as Polymyositis

**DOI:** 10.4269/ajtmh.20-0168

**Published:** 2020-11

**Authors:** Victoria Marie Ferreira Mank, Kevin Francis Brown, Jefferson Roberts

**Affiliations:** 1 Department of Internal Medicine, Tripler Army Medical Center, Honolulu, Hawaii;; 2 Department of Rheumatology, Tripler Army Medical Center, Honolulu, Hawaii

**Figure 1.**
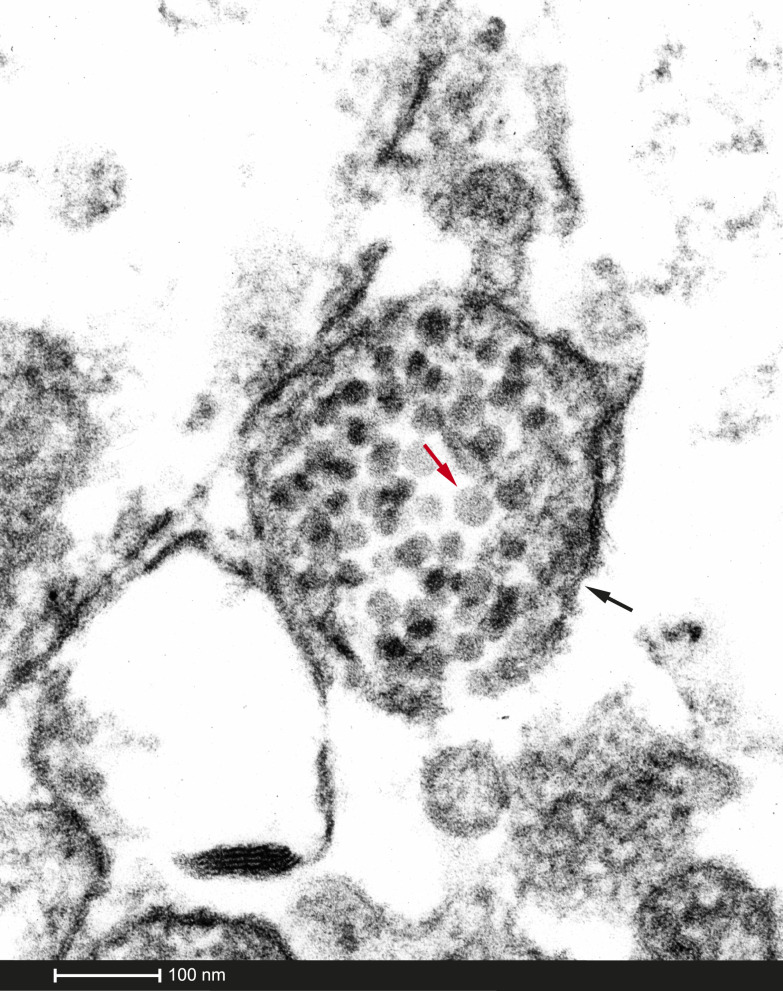
Electron microscopy (EM) imaging (magnification ×302,400): 30–31 nm round to polyhedral viral particles (red arrow), consistent with Picornavirus family inclusive of coxsackievirus, can be seen throughout the muscle biopsy within a spindle-like sheath (black arrow). These viral particles on EM demonstrate direct invasion of muscle tissue by the virus. This figure appears in color at www.ajtmh.org.

A 66-year-old Filipino woman with a history of hyperlipidemia presented with severe pain in her lower extremities causing an inability to ambulate. She reported having diffuse myalgia that was prominent in distal extremities for 1 week before presentation. She discontinued her statin medication, but her pain continued to worsen. Laboratory workup revealed mildly elevated creatine kinase and erythrocyte sedimentation rate. She was initially diagnosed with viral influenza and polymyositis and treated with steroids with no improvement in her worsening proximal muscle weakness. Magnetic resonance imaging of her lower extremities was obtained, which demonstrated diffuse myositis. Viral serologies and titers were obtained, including *Rickettsia typhi*, Epstein–Barr virus, *cytomegalovirus*, *Bartonella henselae* and *Bartonella quintana*, *Toxoplasma gondii*, *Borrelia burgdorferi*, and coxsackievirus B serotypes. All serologies were either negative or low titers, indicative of previous or no exposure, except for coxsackievirus titers. Coxsackievirus B4 IgM titers were 1:80, and coxsackievirus B5 IgM titers were 1:40. Twelve myositis-specific autoantibodies were tested and were negative. Further workup included a muscle biopsy of her right vastus intermedius, which demonstrated moderate type II fiber atrophy. Inflammatory cells within potentially necrotic and regenerating muscle fibers would have indicated polymyositis.^1^ Her muscle biopsy was evaluated under electron microscopy, and viral particles were found strewn throughout her muscle tissue (Figure 1). Viral serologies were reobtained approximately 1 week after the previous laboratory work was collected, and an elevated coxsackievirus B4 IgM titer of > 1:640 and coxsackievirus B5 IgM of 1:80 resulted, confirming the diagnosis of viral myositis. Treatment was switched to supportive care, and she slowly regained full muscle control and strength, with laboratory markers returning to normal within a year.

This case demonstrates the importance of holding a wide differential for muscle weakness to include viral myositis, drug-induced myopathy, metabolic disorders, and inflammatory processes. Clinical manifestations of coxsackievirus may mimic autoimmune and inflammatory conditions.^2^ Studies have begun to show that diagnoses such as chronic fatigue syndrome may actually be a result of previous subclinical inflammatory viral myopathies.^3^ This stresses the importance of maintaining a wide clinical suspicion at initial presentation to potentially prevent progression to other disease processes.
